# Genomic Architecture of Aggression: Rare Copy Number Variants in Intermittent Explosive Disorder

**DOI:** 10.1002/ajmg.b.31225

**Published:** 2011-08-02

**Authors:** Tiffany H Vu, Emil F Coccaro, Evan E Eichler, Santhosh Girirajan

**Affiliations:** 1 Department of Genome Sciences, University of Washington School of MedicineSeattle, Washington; 2 Department of Psychiatry and Behavioral Neuroscience, University of ChicagoChicago, Illinois; 3 Howard Hughes Medical Institute, University of Washington School of MedicineSeattle, Washington

**Keywords:** aggression, array CGH, genomic disorders, segmental duplication, 15q13.3

## Abstract

Copy number variants (CNVs) are known to be associated with complex neuropsychiatric disorders (e.g., schizophrenia and autism) but have not been explored in the isolated features of aggressive behaviors such as intermittent explosive disorder (IED). IED is characterized by recurrent episodes of aggression in which individuals act impulsively and grossly out of proportion from the involved stressors. Previous studies have identified genetic variants in the serotonergic pathway that play a role in susceptibility to this behavior, but additional contributors have not been identified. Therefore, to further delineate possible genetic influences, we investigated CNVs in individuals diagnosed with IED and/or personality disorder (PD). We carried out array comparative genomic hybridization on 113 samples of individuals with isolated features of IED (n = 90) or PD (n = 23). We detected a recurrent 1.35-Mbp deletion on chromosome 1q21.1 in one IED subject and a novel ∼350-kbp deletion on chromosome 16q22.3q23.1 in another IED subject. While five recent reports have suggested the involvement of an ∼1.6-Mbp 15q13.3 deletion in individuals with behavioral problems, particularly aggression, we report an absence of such events in our study of individuals specifically selected for aggression. We did, however, detect a smaller ∼430-kbp 15q13.3 duplication containing *CHRNA7* in one individual with PD. While these results suggest a possible role for rare CNVs in identifying genes underlying IED or PD, further studies on a large number of well-characterized individuals are necessary. © 2011 Wiley-Liss, Inc.

## INTRODUCTION

Recent analyses of copy number variants (CNVs) have revealed associations of genomic changes with a variety of human diseases and disorders. A majority of rare (frequency of <1%) CNVs arise within genomic “hotspots” due to unequal crossover between segmental duplications of high sequence identity [Lupski, 1998; Bailey et al., 2002]. Genome-wide surveys of these hotspots initially detected an enrichment of rare CNVs among individuals with intellectual disability [Sharp et al., 2006; Stankiewicz and Lupski, 2010] and were subsequently identified in individuals with schizophrenia [Walsh et al., 2008], autism [Sebat et al., 2007], obesity [Bochukova et al., 2010; Walters et al., 2010], and epilepsy [Helbig et al., 2009; Mefford and Mulley, 2010].

Aggression is a complex behavior regulated and influenced by an individual's environment, neural brain circuitry, and genetic makeup [Brunner et al., 1993; Manuck et al., 1999; Seroczynski et al., 1999; Volavka, 1999; Davidson et al., 2000; Bevilacqua et al., 2010]. Impulsive aggression as opposed to premeditated aggression is believed to arise from an inability to fully regulate negative emotion and often leads to acts of spontaneous violence [Davidson et al., 2000; Siever, 2008]. This behavior is highly prevalent among violent offenders and has led investigators to explore the possible biological and neurological contributions to the aggression phenotype [Seroczynski et al., 1999; Rajender et al., 2008; Siever, 2008; Craig and Halton, 2009]. Twin and family studies suggest that impulsive acts of aggression are influenced by genetics, with heritability estimates of 44–72% [Miles and Carey, 1997; Seroczynski et al., 1999; Slutske, 2001; Rhee and Waldman, 2002; Yeh et al., 2010]. More than a decade of genetic research using linkage and association studies has identified several candidate genes as contributors to impulsive aggression [Brunner et al., 1993; Manuck et al., 1999; Caspi et al., 2002; Craig and Halton, 2009; Bevilacqua et al., 2010].

Interestingly, aggression, impulsivity, and mood disorders have been reported in many individuals with large genomic rearrangements, particularly those carrying an approximately 1.6-Mbp recurrent deletion on chromosome 15q13.3 spanning segmental duplication breakpoints 4 and 5 (BP4 and BP5). In fact, five separate studies on this particular deletion reported aggressive behaviors in individuals with developmental delay or autism [Ben-Shachar et al., 2009; Miller et al., 2009; Shinawi et al., 2009; van Bon et al., 2009; Cubells et al., 2011]. For example, Ben-Shachar et al. [2009] reported that 9 out of 14 children with the deletion also exhibited some form of abnormal behavior including rage, repeated head banging, and/or attention deficit hyperactivity disorder (ADHD). Behavioral disorders have also been reported in other patients with large CNVs including 1q21.1 cases with intellectual disability [Brunetti-Pierri et al., 2008] and 16p13.11 cases with autism [Ullmann et al., 2007], intellectual disability [Hannes et al., 2009], or schizophrenia [Ingason et al., 2009]. These findings suggest that haploinsufficiency of certain genomic regions affects specific neurobiological pathways predisposing individuals to aggressive and impulsive behavior. However, to date, no study has specifically examined the role of CNVs in isolated aggressive behaviors, such as intermittent explosive disorder (IED).

IED is characterized by recurrent episodes of aggressive behavior and, according to the *Diagnostic and Statistical Manual for Mental Disorders* (DSM-IV) [Association, 1994], cannot be explained by other mental disorders (e.g., borderline personality disorder), the effects of substance use (e.g., medication or drug abuse), or a medical condition (e.g., head trauma). The disorder is estimated to affect approximately 3–4% of the general population, with an early onset age around 14 years [Coccaro et al., 2005; Kessler et al., 2006]. Here we undertook a systematic analysis to assess the contribution of CNVs in 113 individuals diagnosed with IED (n = 90) and/or personality disorder (PD, n = 23) without cognitive deficits. We performed comparative genomic hybridization (CGH), using a custom whole-genome microarray targeted to genomic hotspots, to identify rare, potentially pathogenic CNVs contributing to IED not present in 306 control individuals. For further comparison, we also assessed CNV data from a large set of 5,570 normal adult individuals matched for ethnicity [Consortium, 2008; Itsara et al., 2009].

## MATERIALS AND METHODS

### Subjects

All subjects were physically healthy Caucasian individuals who were systematically evaluated as part of a larger program designed to study the biological correlates of personality traits in human subjects. Study subjects (85 males, 62 females) were recruited by newspaper and public service announcements seeking subjects with and without histories of anger and aggression to take part in medically related studies. Written informed consent, using an IRB-approved consent form, was obtained from all subjects after all procedures were fully explained. The medical health of all subjects was documented by medical history, physical examination, and a variety of clinical laboratory studies, including a urine screen for illicit drugs.

### Diagnostic Assessment

All diagnoses of IED were made as previously described [Coccaro et al., 2009]. A diagnosis of IED was made using the Integrated Research Criteria for IED [IED-IR; Coccaro, 2011], which combines the critical aspects of the DSM-IV and earlier Research Criteria for IED [Coccaro et al., 1998]. Subjects with a life history of bipolar disorder, schizophrenia (or other psychotic disorder), or intellectual disability were excluded from this study. All of the IED-IR subjects (n = 117) met the DSM-IV criteria for at least one lifetime Axis I disorder, and nearly all (n = 107) met DSM-IV criteria for a PD (Table I). Most of the PD subjects (n = 21) met DSM-IV criteria for at least one lifetime Axis I disorder and, by definition, all PD subjects (n = 30) met DSM-IV criteria for a PD (Table I). Subjects from both groups had clear evidence of impaired psychosocial functioning (IED, mean GAF score = 60.5 ± 7.3; PD, mean GAF 65.2 ± 5.3). Aggression was assessed dimensionally (in most, though not all, subjects) using the aggression scales of the Life History of Aggression assessment [Coccaro et al., 1997] and the Buss–Perry Aggression Questionnaire [Buss and Perry, 1992]. Impulsivity was evaluated using the Life History of Impulsive Behavior Questionnaire [Schmidt et al., 2004] and the impulsivity score from the Eysenck Personality Questionnaire-II [Eysenck and Eysenck, 1975].

**TABLE I:** Summary of Lifetime Axis I and Personality Disorder Diagnoses

	IED-IR (n = 117)	PD (n = 30)
Mean GAF score	60.5 ± 7.3	65.2 ± 5.3
Lifetime Axis I disorders	117 (100%)	21 (70%)
Any mood	74 (63.2%)	14 (46.7%)
Major depression	63 (53.8%)	13 (43.3%)
Dysthymia	12 (10.3%)	1 (3.3%)
Depressive NOS^a^	8 (6.8%)	1 (3.3%)
Any anxiety	46 (39.3%)	8 (26.7%)
Phobic	23 (19.7%)	6 (20%)
Non-phobic	46 (39.3%)	7 (23.3%)
Substance use	52 (44.4%)	3 (10%)
Alcoholism	41 (35%)	3 (10%)
Drug dependence	28 (23.9%)	2 (6.7%)
Non-IED impulse control	8 (6.8%)	0 (0%)
Eating	19 (16.2%)	3 (10%)
Adjustment	8 (6.8%)	3 (10%)
Somatoform	5 (4.3%)	0 (0%)
Personality disorders	107 (91.5%)	30 (100%)
Cluster A	21 (17.9%)	2 (6.7%)
Paranoid	20 (17.1%)	1 (3.3%)
Schizoid	1 (0.9%)	1 (3.3%)
Cluster B	62 (53%)	11 (36.7%)
Borderline	46 (39.3%)	7 (23.3%)
Antisocial	21 (17.9%)	0 (0%)
Narcissistic	19 (16.2%)	4 (13.3%)
Histrionic	8 (6.8%)	1 (3.3%)
Cluster C	35 (29.9%)	13 (43.3%)
Obsessive-compulsive	23 (19.7%)	7 (23.3%)
Avoidant	13 (11.1%)	7 (23.3%)
Dependent	2 (1.7%)	1 (3.3%)
PD-NOS^a^	28 (23.9%)	11 (36.7%)

a NOS = not otherwise specified; Note that 113/147 total samples were of good DNA quality and were evaluated for CNVs.

### DNA Samples

DNA was extracted from whole blood by the University of Chicago's Clinical Research Center and the extracted DNA was frozen and stored at −70°C until genotyping. Of the 147 (IED, n = 117; PD, n = 30) individuals evaluated in our research program, 113 samples (IED, n = 90; PD, n = 23) passed DNA quality control and were hybridized using a custom targeted microarray. The control cohort consisted of 306 DNA samples obtained from the Rutgers University Cell and DNA Repository (http://www.rucdr.org). These individuals were ascertained by the National Institute of Mental Health (NIMH) Genetics Initiative [Moldin, 2003] through an online self-report based on the Composite International Diagnostic Instrument Short-Form (CIDI-SF) [Kessler and Ustun, 2004] and screened for major depression, bipolar disorder, and psychosis. Those who did not meet DSM-IV criteria for major depression, who denied a history of bipolar disorder or psychosis, and who reported exclusively European origins were included [Baum et al., 2008; Talati et al., 2008].

### CNV Discovery and Analysis

We utilized the duplication architecture of the human genome to custom design a DNA oligo microarray targeted to the genomic hotspots [Bailey et al., 2002]. This hotspot-targeted microarray consists of 135,000 probes (by Roche NimbleGen) with a median spacing 2.6 kbp in 107 genomic hotspots and 36 kbp in the genomic backbone. Array CGH of patient samples was carried out as described [Selzer et al., 2005] using a single, unaffected male (GM15724, Coriell) as a reference. All arrays were then analyzed by mapping probe coordinates to the human genome assembly build 36 (hg18). Using chromosome-specific means and standard deviations, normalized log intensity ratios for each sample were transformed into *z*-scores. These *z*-scores were then classified as “increased,” “normal,” or “decreased” in copy number using a three-state Hidden Markov Model (HMM). For each sample, HMM state assignments of probes were merged into segments if consecutive probes of the same state were less than 50 kbp apart when merged. If two segments of the same state were separated by an intervening sequence of ≤5 probes and ≤10 kbp, both segments and the intervening sequence were called as a single variant. Subsequently, we automatically filtered and divided putative CNVs based on size, *z*-scores, and probe counts. With these filtering criteria, we were able to thoroughly scan HMM outputs for CNV events and manually check the validity of each call by examining the normalized log intensity ratios across a chromosome.

## RESULTS

A total of 350 CNVs greater than 50 kbp were detected using array CGH among the 113 samples that passed DNA quality control measures (Supplementary Table I). At a similar rate of detection, 1,074 CNVs greater than 50 kbp were seen in our 306 NIMH controls (Supplementary Table II). Overall, there was no difference in the frequency of large CNVs (>500 kbp) between individuals with IED and controls (6/113 vs. 6/306, respectively; Fisher's exact test, *P* = 0.072) assayed on the same microarray platform. After filtering for known copy number polymorphisms, we identified three large rare CNVs: a recurrent 1q21.1 deletion containing *GJA8* [Brunetti-Pierri et al., 2008; Consortium, 2008; Mefford et al., 2008; Stefansson et al., 2008], a novel 16q22.3q23.1 deletion in individuals with IED, and an approximately 430-kbp 15q13.3 duplication containing *CHRNA7* in an individual with PD (Table II). None of these events were observed in our 306 NIMH controls.

**TABLE II:** Clinical Diagnoses of Subjects Carrying Rare CNV Deletions

Subject	Sex	Deletion	Frequency in controls^a^	IED-IR	Other	Axis II	Suicide attempts
Axis I							
1	Female	1q21.1 (1.5 Mbp)^b^	0/5,570; 0/306	Current	MDD, recurrent; AD, partial remission; SD, full remission; PSD, current; ODD, current; ADHD, current	Borderline PD	4
2	Female	16q22.2–q23.1 (350 kbp)^b^	0/5,570; 0/306	Current	CA, past; HD, full remission; GAD, past; ODD, past	Histrionic PD; narcissistic PD	2
3	Male	15q13.3 (430 kbp)^b^	35/5,570; 0/306	N/A	MDD, recurrent; AD, partial remission; SD, full remission; SP, current; GAD, current, ODD, past	Avoidant PD, borderline PD, self-defeating PD	0

MDD, major depressive disorder; AD, alcohol dependence; SD, poly-substance dependence; PSD, post-traumatic stress disorder; ODD, oppositional defiant disorder; ADHD, attention deficit hyperactivity disorder; CA, cannabis abuse; HD, hallucinogen dependence; GAD, generalized anxiety disorder; SP, social phobia; PD, personality disorder.

a Counts from two control groups: 5,570 as previously described by schizophrenia Consortium [2008] and Itsara et al. [2009]; 306 NIMH controls were run using the same NimbleGen microarray design.

b Approximate size of copy number variant; actual breakpoints unknown.

**FIG. 1:**
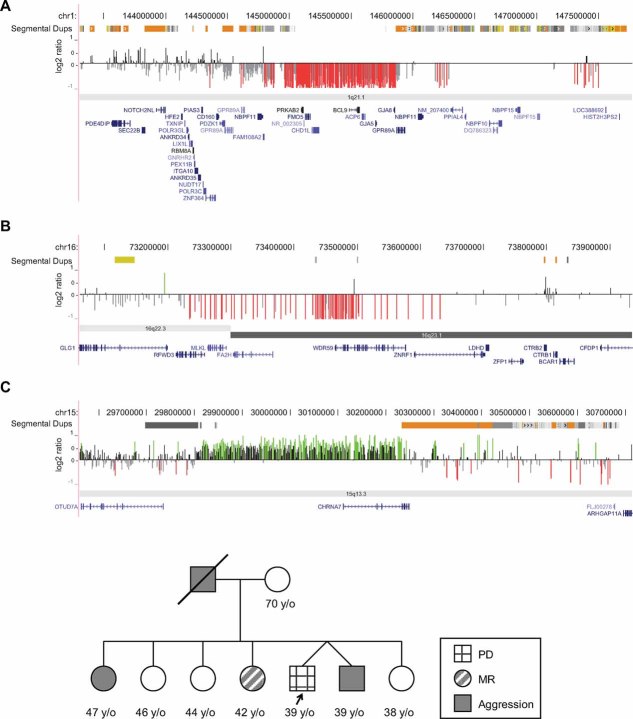
Rare CNVs in IED and PD. **A**: A recurrent ∼1.5-Mbp deletion was detected in 1 of 90 individuals with IED (Subject 1). The undefined breakpoints lie within segmental duplications (green) flanking the deleted region. **B**: A large deletion on 16q22.3–q23.1. This rare deletion was detected in 1 out of 90 subjects diagnosed with IED (Subject 2), which is unreported and remains uncharacterized. The approximately 350-kbp region intersects a total of six genes, including two that are highly expressed in the human nervous system: fatty acid 2-hydroxylase (*FA2H*) and zinc finger and ring finger protein 1 (*ZNRF1*). **C**: A recurrent duplication on 15q13.3. This duplication was identified in 1 out of 23 subjects diagnosed with PD (Subject 3). Currently, it is unclear whether this duplication is pathogenic. The lower panel shows a family pedigree of Subject 3 (arrow) who was diagnosed with PD (checkered) and family members with a history of aggression (solid) and mental retardation (diagonal) are also depicted.

The ∼1.35-Mbp deletion (chr1: 144,979,471–145,863,720) on chromosome 1q21.1 (Fig. 1A) was detected in Subject 1, who had features of IED as well as ADHD, borderline personality disorder, and a history of major depressive disorder, alcohol dependence, and poly-substance dependence. Family history suggests problems with anger and aggression in the father but not in the mother. The subject's older brother has a history of anger and aggression, depression, and a possible diagnosis of schizophrenia while the older sister has had individual and family therapy for emotional problems and difficulties with anger. The subject's younger sister has a history of anorexia, substance abuse, and has also been in therapy. The subject also reported experiencing physical and sexual abuse for herself and her siblings from their father. Detailed evaluation showed that this individual has had at least three verbal arguments/temper tantrums per week over the past 23 years (since 18 years old). There is also a history of more than 20 juvenile arrests for physical fighting albeit without convictions. Outbursts were described to involve snapping, yelling, swearing, and physical fighting, sometimes escalating to property damage. The greatest period in which the subject reported property damage occurred at a frequency of two times per month between the ages of 18–26. For about a decade, between the ages of 19–30 years old, the subject reported involvement in physical assault at about two times per week at the highest frequency. One extreme episode resulted in a week spent in jail and a requirement to take anger management class. In addition to these outbursts, the subject reported multiple attempts at suicide (Supplementary Material).

The ∼350-kbp deletion we detected on chromosome 16q22.3q23.1 (chr16: 73,290,945–73,626,825) in Subject 2, encompasses five RefSeq genes including *FA2H* and *ZNRF1* (Fig. 1B). This event was not seen in any of the 5,876 controls analyzed in our study, suggesting that this event is novel. There is no history of anger and aggression or substance abuse in the parents. However, anger problems were reported in the subject's sister. There is no childhood history of physical or sexual abuse. Subject 2 typically engaged in two verbal arguments per week for the past 6 years (since 18 years old). These outbursts were described to include yelling, insulting, and swearing at others. This individual recalled one incident of property damage and one episode of physical aggression; however, both events were minor and had no legal consequences. Aggressive episodes occurred while the subject was sober and were spontaneous in nature. This subject was also diagnosed with histrionic and narcissistic PD along with a history of drug abuse, hallucinogen dependence, generalized anxiety disorder, and oppositional defiant disorder (Supplementary Material).

While we did not identify the larger 15q13.3 (BP4–BP5) deletion previously associated with aggressive behaviors, we detected an ∼430-kbp duplication nested within the BP4–BP5 locus and encompassing *CHRNA7* in Subject 3 (Fig. 1C). Subject 3 does not have IED but was diagnosed with avoidant, borderline, and self-defeating PDs, social phobia, generalized anxiety disorder, and a history of depression, alcohol dependence, drug dependence, and oppositional defiant disorder. Further evaluation of family members indicated a history of aggression in a twin brother and a sister and intellectual disability in another sister (Fig. 1C). The father was described to have problems with aggression and alcohol. DNA samples from family members were not available for further testing so the carrier status of this 15q13.3 duplication in other family members is not known.

## DISCUSSION

In this study we aimed to determine whether rare pathogenic CNVs are a predisposing factor for impulsive aggressive behavior, due to growing evidence that genomic architecture is highly significant to human biology and disease [Marques-Bonet et al., 2009; Mefford and Eichler, 2009]. We were particularly interested to see if 15q13.3 (BP4–BP5) deletions would be enriched in our subjects. Previous reports have emphasized the presence of neuropsychiatric disorders and neurobehavioral problems in patients with this particular alteration on chromosome 15q13.3 (Table II). We did not find any 15q13.3 (BP4–BP5) deletions in our cohort of 113 individuals; however, we recognize that the small sample size of our study cohort limits the power to detect this rare rearrangement. In a study of patients with schizophrenia, this deletion was found in 9/3,391 patients as opposed to 0/3,181 controls [Consortium, 2008]. Our results, therefore, do not completely rule out the possible association of this deletion with aggressive and impulsive behavior.

While the larger 15q13.3 deletion was not observed among IED subjects, we did find a smaller duplication nested within BP4 and BP5 in one subject with PD. This duplication was observed in 35/5,570 and 0/306 of our two control groups. While a heterozygous loss of the region is associated with variable neurodevelopmental phenotypes [Shinawi et al., 2009], the clinical significance of a gain is still undetermined. Recently, Szafranski et al. [2010] attempted to determine whether these duplications are benign or pathogenic by investigating this alteration in 59 patients with a range of phenotypes. After mapping the breakpoints for each event, they were able to determine two classes of large ∼1.5-Mbp (BP4–BP5) duplications (n = 4) and five classes of smaller duplications (n = 55), similar to that found in Subject 3. Six of eleven index patients with a smaller duplication, who were diagnosed with developmental delay or intellectual disability, also had some neuropsychiatric issues, including bipolar disorder, anxiety disorder, disruptive behavior disorder, and severe pica. While these observations suggest the possible association of the smaller 15q13.3 duplications with neurocognitive and neuropsychiatric disorders, the comparable detection rates of this CNV in controls argues otherwise. It is possible, however, that control cohorts were not screened for mild neuropsychiatric behaviors and confound current estimates. Subject 3 in our study is affected by a number of PDs and our findings support the need for more robust studies of 15q13.3 duplications as a susceptibility locus to milder neurobehavioral disorders (Supplementary Material).

The 1q21.1 deletion we identified in Subject 1 has been associated with variable neurodevelopmental phenotypes and is enriched in affected populations compared to controls. The recurrent 1.35-Mbp deletion has been detected in 25/5,218 unrelated patients with mental retardation, microcephaly, cardiac abnormalities, or cataracts [Mefford et al., 2008] and 10/3,391 patients with schizophrenia [Consortium, 2008]. In comparison, the event was only seen in 1/7,918 total controls. Behavioral abnormalities have also been observed in patients with this deletion. Brunetti-Pierri et al. [2008] describe anxiety/depression, antisocial behavior, and aggression in 3 out of 21 probands with dysmorphic features. A loss at this particular locus may be associated with a broader range of phenotypes than is currently described, possibly influencing the behaviors seen in our subject; however, a greater number of samples must be analyzed to draw a definitive conclusion (Table III).

**TABLE III:** Summary of Studies Reporting Aggressive Behaviors in Individuals With Rare CNVs

CNV	Ascertainment	Total cases	(+) Aggressive and/or impulsive behavior	Study
15q13.3 (del)	Developmental delay, epilepsy^a^	10	1	Shinawi et al. [2009]
15q13.3 (del)	Developmental delay, epilepsy, autism spectrum disorder	5	5	Miller et al. [2009]
15q13.3 (del)	Developmental delay, autism spectrum disorder^b^	14	9	van Bon et al. [2009]
1q21.1 (del)	Congenital heart defects, developmental delay, schizophrenia^c^	21	1	Brunetti-Pierri et al. [2008]
16p13.1 (del/dup)	Intellectual disability, multiple congenital abnormalities	13	4	Hannes et al. [2009]
16p13.1 (del/dup)	Intellectual disability, autism	7	2	Ullmann et al. [2007]

a Ten individuals from four unrelated families.

b Fourteen children from 12 unrelated families.

c Two probands also described to have behavioral problems.

The second rare CNV identified in this study, a deletion on chromosome 16q22.3q23.1, is currently uncharacterized and was not seen in 5,876 controls. Three RefSeq genes (*MLKL*, *FA2H*, and *WDR59*) are completely deleted by this event, and two (*RFWD3* and *ZNRF1*) are partially deleted. Notably, fatty acid 2-hydroxylase (*FA2H*) and zinc finger and ring finger 1 (*ZNRF1*) are two genes involved in the formation and insulation of neuronal axons. Specifically, *FA2H* contributes to myelin formation and *ZNRF1* plays a role in Schwann cell repair; both are highly expressed in nervous tissue [Araki et al., 2001; Alderson et al., 2004]. Reduced white matter integrity (myelinated axons in the brain) has been reported in schizophrenics with significant histories of aggression [Hoptman et al., 2002] and in subjects with IED-IR [Coccaro, unpublished data]. It is possible that the disruption of *FA2H* and *ZNRF1* creates a deficiency in neural maintenance and repair, predisposing an individual to deleterious injuries that can result in neuropsychiatric disorders such as IED.

Our results highlight the possible role of genomic rearrangements in impulsive aggressive behavior and PDs. However, the complex association of these rearrangements with a variety of phenotypes suggests that other factors in addition to CNVs are influencing what ultimately manifests in individuals [Girirajan et al., 2010]. In IED, environmental effects and individual life experiences seem to play a vital role in whether individuals with predisposing genetic variants actually present with clinical features. It may be that impulsive aggression is a behavior that frequently co-occurs with genomic disorders as a result of underlying biological processes that are disrupted. In future work, systematic examination of psychosocial factors, sequencing top priority genes, or carrying out exome or whole-genome sequencing of affected individuals, in addition to CNV analysis, may provide insight into the genetic variability that contributes to IED.
