# Supplementary material for: Genomic Architecture of Aggression: Rare Copy Number Variants in Intermittent Explosive Disorder
Source: Am J Med Genet B Neuropsychiatr Genet. 2011 Aug 2;156(7):808–16. doi: 10.1002/ajmg.b.31225 (PMC3168586; doi:10.1002/ajmg.b.31225)
Supplement: Supplementary file 1 [file ajmg0156-0808-SD1.doc]

Supplementary Material

Genomic architecture of aggression: Rare copy number variants in intermittent explosive disorder

Tiffany Vu, Emil F. Coccaro, Evan E. Eichler, Santhosh Girirajan

Table of Contents

[Clinical findings in Subject 1 2](#__RefHeading___Toc295565633)

[Clinical findings in Subject 2 9](#__RefHeading___Toc295565634)

[Clinical findings in Subject 3 14](#__RefHeading___Toc295565635)

# Clinical findings in Subject 1

**Axis I Disorders:**

Mood Disorders: The subject reported several periods of depressed mood for most of the day nearly every day for at least 2 weeks. She feels that the most severe episode was sometime between 1995 and 1998 after the breakup of a relationship. Her depressed mood lasted about 3-4 weeks, and she lost interest in doing things she normally enjoyed (doing activities with family and friends). She endorsed the following symptoms of depression during that time: weight loss of 15 lbs and decreased appetite (she reported having about 50% of her normal appetite); sleeping about 4 hrs per night when 6 hrs was normal for her; psychomotor retardation (to the point that family and friends noticed it); having “zero energy” and feeling tired all of the time; feeling worthless, like “a failure and a loser;” difficulty concentrating and making decisions (thus interfering with work, her social life, and family); and recurrent thoughts of her own death. These symptoms caused significant impairment in her social relationships and negatively affected her work performance. These symptoms do not appear to be the result of a medication or another mental disorder. The subject was treated with Wellbutrin for 6 months following this depressive period. She reported several other episodes of depression in which her symptoms were not as severe (June 2003- following the breakup of a 4-year relationship (she was still depressed in Oct 2003 at the time of her initial screen for the study) and 1986-at the time of her last suicide attempt and during a time when she was drinking and drugging heavily). **Impression**: A diagnosis of Major Depressive Disorder.

Substance Use Disorders, Alcohol: The subject reported over the past 6 months drinking about 2-3 glasses of wine per week. She has admitted to drunk driving once in the past 6 mos. No other symptoms indicative of current abuse were endorsed. In the past (between 1986 and 1999 for about 6-7 yrs total) she was drinking 5-7 days per week, having about 6 drinks per occasion. She reported drinking much more than she had intended “all of the time”. She was spending a great deal of time being drunk or hung-over (every day of the week). She reports having “hundreds” of blackouts when drinking and continuing to drink anyway. The subject reports developing tolerance, needing to drink larger amounts over time (although she can’t remember how much more) and markedly diminished effect with continued use of the same amount of alcohol. She reported several withdrawal symptoms when trying to cut down on her drinking: sweating, hand shakes, feeling agitated and feeling anxious. She was 18 y/o when she first started having these symptoms, and it has been 5-6 yrs since she has been drinking at this rate or had any of these problems. Her friends were objecting to her drinking and eventually got her to go to AA (around age 26). While going to AA, there was a period of 1.5 yrs when she abstained from alcohol completely. **Impression:** Alcohol Dependence, Past, in Sustained Partial Remission is the suggested diagnosis. She also meets criteria for NIMH RDC Alcoholism, Past.

Substance Use Disorders, Non-Alcohol: The subject reports using marijuana occasionally for about 10 yrs, but never more than 10 times in one month. She reports using prescription drugs (including Vikadin, Darvon, Flexiral, Valium, Xanax, and Halcion) when not prescribed and becoming hooked on them. This started in her early teens; she reported prescription drugs being readily available in her mother’s medicine cabinet (“there was always something around to take”). Her drug use became a more regular thing from age 18-35. She used cocaine in the 1990’s for about 3-4 yrs (she was between 26-36 y/o). She reported using speed/crystal meth 3 times per week for about 2 years (between ages 28-35). During this time, no single substance predominated. Subject reported “It didn’t matter what I was taking as long as I could get high”. She often ended up using much more than she had planned to. She was spending a great deal of time using and recovering from her substance use. She often spent the weekend just doing drugs when she normally would have been doing other things; therefore, recreational activities were given up or reduced. She felt that the drugs caused/contributed to depression, but she kept using anyway. She reported developing a tolerance (over time she felt like she was using about 50% more). **Impression:** Poly-substance Dependence, Past, in Sustained Full Remission is the suggested diagnosis.

Anxiety Disorders: The subject reported having about three panic attacks, two of which occurred in Sept 2003 after the break-up of a relationship. These two were situational and during a depressive episode. The subject could not remember any details about the other panic attack, but it was possibly also after the breakup of a relationship. Because these panic attacks are not unexpected or recurrent, no diagnosis is suggested. The subject reported several traumatic things happening to her: two bad auto accidents and sexual abuse by her grandmother (at age 5) and by her brother (when she was 10-12). She identified the incest as the most severe trauma she experienced. During the abuse, she felt helpless and afraid. She reported the following re-experiencing symptoms after the incest: distressing recollections (particularly in sexual situations with partners), recurring dreams about the abuse, feeling as if the trauma was recurring (particularly with men), getting extremely upset when something reminded her of the trauma (“flying off the handle” and getting angry), and physiological reactivity upon exposure to cues that resemble the event (heart pounding or racing, feeling very nervous). She reported the following avoidance symptoms after the trauma: efforts to avoid thinking or talking about the abuse; efforts to avoid activities, places, or people that arose recollections (by not letting anyone touch her in intimate relationships); inability to recall an important aspect of the trauma; diminished interest or participation in activities; feeling detached or estranged from others; restricted range of affect (feeling numb and that she hated everyone); and having a sense of a foreshortened future. The subject reported the following symptoms of increased arousal after the trauma: irritability and outbursts of anger, hypervigilance, and exaggerated startle response. She can’t remember exactly how old she was when she began to have these symptoms but thinks it was probably around age 5. She has not had the symptoms since age 34. **Impression:** Post-Traumatic Stress Disorder is the suggested diagnosis.

Suicidality/Self-injurious Behavior: The subject has made four suicide attempts, the first one at age 19, 2 in her early 20s, and one at age 26. They all involved taking an unknown amount of pills (Halcion, Xanax- between 10 and 50 pills total). The last three attempts resulted in hospitalization, with the subject staying for 1-2 days. She describes all of the attempts as “cries for help.” In all of the instances, she knew that someone would find her and/or intervene. She reported that the attempts didn’t occur when she was drinking or taking drugs, however, she was experiencing “mild” depression at the time. The subject has also engaged in self-injurious behaviors (predominately cutting on her arms and occasionally head-banging). She reported this first occurring between ages 13/15, and the last time she engaged in cutting was in 2000. She had a well-defined plan to cut herself once within the last year (after the breakup of her 4 yr relationship), but she did not carry it out. She has sustained some scars from the superficial cuts and has had some bruising and pain on her head from the head banging, but she never had to seek medical attention.

Aggressive Disorders: LHA=23, LHWA=81, LHEA=85 She reported that these began at age 2 or 3 and are ongoing. For the past 23 yrs (since age 18), the subject has engaged in about 3 verbal arguments/temper tantrums per week, and this represents the greatest reported period of frequency. She described three verbal outbursts: one with an ex-girlfriend involving snapping, yelling, swearing, yelling, and escalating in to property damage (the police were called); one with a date involving raised voices and insults, and one with her sister involving swearing, yelling, and escalating into a physical fight. The subject reported 50 incidents of property damage beginning age 18, all of which were worth more than $50 or had significant sentimental value. There have been no instances of property damage in the past 9 mos, but there were 2 within the first 3 months of last year. The period of greatest frequency was 2 times per month when the subject was between ages 18-26. The subject described 3 incidents: throwing an irreplaceable sculpture out of the window after an argument with her ex-partner (the police were called); kicking the bed, turning over the desk, breaking things, and pulling the phone out of the wall so her girlfriend couldn’t call the police; and using a baseball bat to break pictures, lamps, the bed and other objects after seeing the Rodney King incident on TV. Subject reported that she has engaged in about 250 physical assaults (125 of them causing minor injury, 125 causing no injury) in her adult life. These outbursts began at age 6 or 7 and there have been none in the last year (age 40 was the last time she engaged in one). At their greatest frequency, these assaults were occurring 2 times per week for about 10 yrs (between ages 19-30). She reported that her most severe physical outburst was with an ex-girlfriend, in which she hit her with a closed fist, kicked her, pushed her down, pulled her hair, and slapped her. As a result, the subject was arrested, spent one week in jail, and took anger management classes. The 2 other incidents were with another ex-girlfriend and a friend and both involved pushing and shoving. The degree of her aggressiveness is grossly out of proportion to the stressors, is impulsive in nature, and causes marked distress (problems in family, friend, and romantic relationships). The aggressive behavior does not appear to be better accounted for by another mental disorder or substance. *Suggested diagnoses are IED 4+, Level 3, current; IED-R, current; and IED-IR, current.*

Impulse Control Disorders: The subject reported having a problem with recurrent gambling a few times (about one time every 3 years for the past 15 years she spends more than she should). After losing money, she has returned to try and make up for her losses several times (e.g. not having enough money to pay for hotel or food, then finding $20 and gambling again until she had enough to pay the hotel bills). She lies to family members and others to try to minimize her gambling almost every time she gambles. Her gambling started at age 18 and is current (last time was one week ago). She denied all other symptoms of pathological gambling, so no diagnosis is suggested.

Childhood Disorders: The subject reported that during childhood and adolescence she was often defiant, hostile, and negativistic towards adults. She endorsed the following symptoms during that time: often losing her temper, often arguing with adults, often refusing to do what adults told her to do, often deliberately annoying people (and “getting a kick out of it”), often being irritable/touchy and easily annoyed by others, often being angry and resentful, and often being spiteful and vindictive. These behaviors and attitudes caused problems for her at home and school and lasted from around age 8 until now (they are ongoing). **Impression:** Oppositional Defiant Disorder is the suggested diagnosis.

The subject reported that during childhood, before adolescence, she had problems paying attention to details and had a short focus, was easily distracted, was “always” fidgety and squirmed in her seat, had trouble staying in her seat at home, was “driven by a motor,” ran around and climbed on things at inappropriate times, got into trouble for blurting out answers before questions were completed, had problems organizing things, didn’t like tasks that required a lot of concentration or focus, often forgot things, was “very loud” and had a hard time playing quietly, had difficulty waiting her turn when playing (she often pushed her way to the front of the line), and often interrupted or intruded on others both verbally and physically. She has had these problems for as long as she can remember (probably before age 7), and they are ongoing. These behaviors caused problems for her at home and at school. **Impression:** Attention-Deficit Hyperactivity Disorder, Predominately Hyperactive-Impulsive Type is the suggested diagnosis.

**Axis II Disorders:**

Subject meets General Criteria for Personality Disorder. She endorsed 20 traits as pathological.

Paranoid PD (2): *Reads hidden demeaning or threatening meanings into benign remarks or events*: She says that she tends to “over-analyze” and take offense at things that weren’t meant to be critical. Both friends and romantic partners have said that she is “overly sensitive” in relationships. *Persistently bears grudges*: She would stay angry with someone that forgets her birthday for a couple of months, but it might come up again later. She tends to hold grudges, and right now she hasn’t forgiven her ex-girlfriend of 4 yrs (the most recent breakup). She also held a grudge against her brother for many years.

Antisocial PD (2): *Failure to conform to social norms with respect to lawful behavior as indicated by repeatedly performing acts that are grounds for arrest:* She reports being arrested 4 times (3 DUIs and 1 battery charge), having a warrant out for her arrest for tickets, and being “drunk and disorderly” many times. *Irritability and aggressiveness, as indicated by repeated physical fights or attacks*: See section on IED.

Borderline PD (5): *Frantic efforts to avoid abandonment*: She reports being frequently upset that someone might leave her, and she “tries everything” to keep them from leaving, “whatever it takes”. She has also made threats of suicide and has made several suicide attempts. *A pattern of unstable and intense interpersonal relationships characterized by alternating between extremes of idealization and devaluation*: This describes “all” of her romantic relationships but not with friends. *Recurrent suicidal behavior, gestures, or threats, or self-mutilating behavior:* See section on Suicidality/Self Injurious Behavior. *Affective instability/marked reactivity of mood*: She reports strong mood swings, often daily, with several different moods within the same day changing in a matter of hrs. *Inappropriate, intense anger or difficulty controlling anger*: See section on IED.

Histrionic PD (3): *Self-dramatization, theatricality, and exaggerated expression of emotion:* She “wears her emotions on her sleeve” (and she feels that this is true for the whole range of emotions). Close friends and family have described her as “too emotional.” *Considers relationships to be more intimate than they actually are*: She feels that she does this about 60% of the time.

Dependent PD (1): *Urgently seeks another relationship as a source of care and support when a close relationship ends*: She reports that this has been a pattern for her in the past.

Self-Defeating PD (2): *Is uninterested in or rejects people who consistently treat her well:* She reports having trouble maintaining relationships with people who treat her better than she thinks she deserves more than half the time (in all of her romantic relationships, and with some others). *Engages in excessive self-sacrifice that is unsolicited by the intended recipient:* She recognizes this as a pattern for her, often treating large groups of people to things (beyond her means). Depressive PD (3): *Self-concept centers around beliefs of inadequacy, worthlessness, and low self-esteem:* She reports always having struggled with this, about 75% of the time. *Is critical, blaming, and derogatory toward self:* She has always been one to put herself down and criticize herself; this is a pattern for her. *Is prone to felling guilty or remorseful*: This is also a pattern for her; she feels guilty about 75% of the time.

Negativistic PD (2): *Is sullen and argumentative:* About 75% of the time, it seems like every discussion turns into an argument, mostly with romantic partners. *Alternates* Subject ##740468 8

*between hostile defiance/contrition*: About 75% of the time, she feels very guilty and takes it back after telling someone off.

**Impression:** Borderline Personality Disorder is the suggested diagnosis.

FINAL DIAGNOSES DSM-IV: Axis I: 296.36 Major Depressive Disorder, Recurrent, in Full Remission 303.90 Alcohol Dependence, in Sustained Partial Remission NIMH RDC Alcoholism, Past 304.80 Poly-substance Dependence, in Sustained Full Remission 309.81 Posttraumatic Stress Disorder IED 4+, Level 3, Current IED-R, Current IED-IR, Current 313.81 Oppositional Defiant Disorder 314.01 Attention-Deficit/Hyperactivity Disorder, Predominately Hyperactive-Impulsive Type Axis II: 301.83 Borderline Personality Disorder GAF (average past year): 40

# Clinical findings in Subject 2

**Axis I Disorders:**

Substance Use Disorders: Subject reported smoking one marijuana joint 3 times a week from ages 15-18. She denied all symptoms of dependence. She stated her use distracted her from school, affecting her grades; she denied all other symptoms of abuse. She denied smoking marijuana since age 18. **Cannabis Abuse, Past is suggested.** She reported taking 1 tab of LSD 2-3 times per month from age 16-17. She stated she took LSD in larger amounts and over a longer period of time than planned, and that she made 3-4 unsuccessful attempts to cut down. It took her several hours to get back to normal after getting high, and made her sick at school. She continued using LSD despite the fact that it made her more anxious than usual. When she quit or cut down, she experienced withdrawal symptoms (vomited, was paranoid and suspicious). She denied using LSD since age 17. **Impression**: Hallucinogen Dependence, With Physiological Dependence, In Sustained Full Remission is suggested.

Anxiety Disorders: Subject reported having several panic attacks 3 years ago while planning her wedding. She stated they happened out of the blue, while she was watching TV or “just sitting” at home. She denied worrying about the implications of the attacks, or about having additional attacks for more than a few days after they occurred. She denied changing any behaviors because of the attacks. As per report, she went to the emergency room during one of the attacks, and when she found out nothing was physically wrong with her, she reportedly felt “ok.” She began taking Xanax after her trip to the emergency room. **No diagnosis is suggested.** Subject reported worrying more days than not for most of 2003. She worried about her job, her friends and social situation, planning her wedding, her relationship with her in-laws, and about not getting enough sleep. She stated she worried about “everything and nothing in particular,” and “felt pulled in different directions.” As per report, she found it difficult to control the worry. She reported experiencing the following for over 6 months: feeling restless, keyed up and on edge, having difficulty concentrating, being irritable, and having difficulty falling or staying asleep. **Impression**: Generalized Anxiety Disorder, Past is suggested.

Childhood Disorders: Subject reported being more defiant, hostile and negativistic than most kids between the ages of 16-18. During this time, she often lost her temper, often argued with adults, often refused to do what adults told her to do, was often touchy or easily annoyed by others, and was often angry and resentful. These behaviors and attitudes caused problems for her at home, school and with friends. **Impression**: Oppositional Defiant Disorder, Past, is suggested.

Suicidality/Self-injurious behavior: **Suicide Attempts = 2.** The subject reported swallowing half-a-bottle of ibuprofen at age 13 and age 16. She reported doing this to gain attention, and denied having real intent to die. Her parents “had an idea” that she did this, but she denied facing any consequences other than being sick and embarrassed. She denied telling anyone about the attempts, or seeking medical attention. **Self-injurious Behaviors = 0.** The subject denied any self-injurious behavior, answering no when asked on the SIBHF if she has ever tried to physically hurt herself or purposely done anything that physically harmed her. Aggressive Disorders: LHA/A=18 Subject reported that she has engaged in approximately 620 verbal arguments and 2 temper tantrums since age 18. She stated her verbal outbursts began around age 13, and is ongoing. The subject reported having 2 verbal arguments per week for the past 6 years. She gave an example of a typical outburst. She stated her husband will either argue against her opinions, or not help around the house enough, and she will yell, insult him and swear at him. She denied any consequences for this behavior, and stated they usually “talk it out.” This participant reported engaging in one instance of property destruction when she was 18. She was angry with a friend and uprooted a potted plant outside of an apartment building. She was warned by police not to do this again, and faced no other consequences. The subject reported engaging in 1 episode of physical aggression since age 18. This occurred when she was 19 years old. She was moving, and her husband (then boyfriend) didn’t help her. She began to yell at him and pushed and shoved him. She denied he was injured in any way, and denied any consequences. Subject reported that her outbursts occur when sober. Subject reported that her outbursts are spontaneous, and denied that they are done for any gain. She stated she has been distressed after outbursts, because they cause problems in her marriage. **IED-IR, Current, and IED-R, Current are the suggested diagnoses.**

**Axis II Disorders:** Subject meets General Criteria for Personality Disorder. She endorsed 20 traits as pathological.

Paranoid PD (1): *Perceives attacks on her character that are not apparent to others and is quick to react angrily or counterattack.* Subject stated that since she miscarried Subject #1000719 4

one year ago, her mother-in-law makes comments about single people who have children. Subject feels as if her mother-in-law is really asking her why she doesn’t have kids, when it would be easier for her to raise children because she is married. She reacts by “getting pissed off.” Her husband tells her she’s being too sensitive.

Borderline PD (3): 1). *A pattern of unstable and intense interpersonal relationships characterized by alternating between extremes of idealization and devaluation.* As per report, relationships with her husband and best friends are marked by a lot of arguing. She stated she often switches from loving and admiring people at one time, to hating them at another time. 2). *Recurrent suicidal behavior.* Subject threatens to kill herself in ¼ of the arguments she gets into. She attempted to kill herself at age 13 and at age 16. 3). *Inappropriate, intense anger, or difficulty controlling anger.* See section on IED. Subject reported feeling angry 50% of the time.

Histrionic PD (5): 1). *Is uncomfortable in situations in which she is not the center of attention.* Subject tries to be the center of attention, even if she “has to do something drastic.” She reported feeling depressed and lonely when not in the center of things. 2). *Interaction with others is often characterized by inappropriate sexually seductive behavior.* Subject reported having a reputation for being a flirt. She said she’s “comfortable socially.” She reported that before she was married, if she saw someone she was attracted to she would “gravitate toward them even if they were like, ‘Who are you?’” 3). *Consistently uses physical appearance to draw attention to self.* Subject uses her physical appearance to get people’s attention by wearing tighter clothes. She is disappointed when people don’t notice how she looks half of the time. During the screen, subject wore a halter-top, even though it was cold outside. 4). *Shows self-dramatization, theatricality and exaggerated expression of emotion.* As per report, subject shows her emotions more than most people. She stated her husband often gets embarrassed by the way she reacts to things (sporting events, loud laughter.) Her husband and in-laws make comments that she shows too much emotion. 5). *Is suggestible.* Subject said she “sometimes” suddenly gets headaches or feels sick when other people complain about feeling this way. She said she “can’t put a number on” how often this happens. **Histrionic Personality Disorder is suggested.**

Narcissistic PD (5): 1). *Is preoccupied with fantasies of unlimited success, beauty, etc.* Subject reported spending ½ of each day daydreaming about being thinner, having the “right proportions” and thinking what she could be doing if her parents had paid for her education. 2). *Believes she is “special” and unique and can only be understood by other special people.* Subject acknowledged feeling as if she is so creative and unique that she has a hard time finding people like herself to share things with. She stated that a person who is “open to new ideas, outgoing, into different cultures and political views, who is artistic and interested in the same sports” is able to understand her and be a good friend. 3). *Requires excessive admiration.* Subject reported feeling empty and hurt 30-40% of the time because she doesn’t get the praise and admiration she thinks she deserves. 4). *Is interpersonally exploitative.* Subject reported manipulating men by flirting with them to get what she wanted. She reported taking advantage of them because it was the only way to get what she needed or deserved. Subject stated this

behavior ceased 2 years ago when she got married. 5). *Shows arrogant, haughty behaviors or attitudes.* Others tell her she has an attitude problem and say she “thinks too highly” of herself. **Narcissistic Personality Disorder is suggested.**

Obsessive-Compulsive PD (1): *Shows rigidity and stubbornness.* Others describe her as being stubborn and set in her ways because she has “set opinions, and high standards of how life in general should be.”

Self-Defeating PD (1): *Rejects or renders ineffective the attempts of others to help her.* Subject stated it is hard for her to accept help from others, even when she needs it. She reported rejecting their help 60% of the time. For example, she refused help with planning or paying for her wedding, even though it made her extremely anxious. She also has refused help with her anger problem. She stated when people offer to help her, she tells them it won’t work because she’s “too busy and it’s pointless.”

Depressive PD (2): 1). *Is negativistic, critical and judgmental toward others.* Subject is quick to criticize the character of other people over 50% of the time. 2). *Is prone to feeling guilty or remorseful.* Subject reported being quick to feel guilty or responsible whenever anything goes wrong over 50% of the time. She feels this way even if others don’t blame her. She stated other people tell her she’s too quick to apologize, even when it’s not her fault, and she agrees. Negativistic PD (2): 1). *Expresses envy and resentment toward those apparently more fortunate.* Subject often talks to her husband about how unfair it is that others have more money than they do, and that she had a miscarriage when other people haven’t. 2). Subject reported that when she tells someone off, she feels very guilty and takes it all back 50% of the time.

FINAL DIAGNOSES DSM-IV: Axis I: 305.20 Cannabis Abuse, Past 304.50 Hallucinogen Dependence, With Physiological Dependence, In Sustained Full Remission 300.02 Generalized Anxiety Disorder, Past 313.81 Oppositional Defiant Disorder, Past 312.34 IED-R, Current

IED-IR, Current Axis II:301.50 Histrionic Personality Disorder 301.81 Narcissistic Personality Disorder GAF (average past year): 53 IED-IR: Positive MAJOR MOOD DISORDERS: Negative NIMH ALCOHOLISM: Negative DSM DRUG DEPENDENCE: Positive SUICIDAL BEHAVIOR: Positive SELF-INJURIOUS BEHAVIOR: Negative

# Clinical findings in Subject 3

**Axis I Disorders:**

Mood Disorders: The subject reported depressed mood for most of the day nearly every day since November. He reported decreased appetite (and sometimes having to consciously make himself eat) and weight loss of a couple of lbs; sleep disturbance alternating between getting hardly any sleep and getting more sleep (waking in the night, unable to go back to sleep or going to bed right when he gets home from work; diminished ability to think or concentrate (hard to stay focused, worse than normal), and thoughts of his own death (no specific plan and a “strong instinctual urge not to do it”). At a sub clinical level, he reported psychomotor agitation and feeling tired emotionally but not physically. He feels that these symptoms have caused distress for him. He recently took some time off work to “think about things” and go to a meditation seminar and start participation in this study. Subject meets criteria for a current major depressive episode. Subject has experienced 2 other periods of depressed mood for most of the day nearly every day for at least 2 weeks: at age 21 during his senior yr of college and at the end of his marriage. He reported that the period at age 21/22 lasted approximately 9 months (his entire senior yr). He reported depressed mood, anhedonia (withdrawing from friends), insomnia (getting about 4 hrs per night when 7 was normal, waking frequently), loss of energy nearly every day, diminished ability to think or concentrate (“I couldn’t study at all”), and thoughts of his own death (though he wasn’t actively considering suicide- the thought might have crossed his mind). He reported the following at a sub clinical level: weight gain of maybe between 2-10 lbs over the course of that yr), alternating slight psychomotor retardation and agitation, and low self-esteem (not feeling good about himself but not quite worthless). This interfered with school and his social life (“I shut down”). He went to the college counseling center to talk to someone; this was very helpful and “got him through the yr.” **Major Depressive Disorder, Recurrent is the suggested diagnosis.**

Subject reported a 2 yr period of feeling depressed more than half the time during the last 2 yrs of college (his junior and senior yrs). However, one yr into this, he experienced a major depressive episode. **No diagnosis is suggested**.

Substance Use Disorders, Alcohol: The subject currently drinks 1-2 days per week, 2-3 drinks per occasion. He denied all symptoms of current abuse; he reported that since he knows alcohol is a depressant, he intentionally stays away from it when he’s already feeling depressed. He reported cutting down over 6 months ago. He has been drinking from age 11-now; he identified his period of heaviest drinking as in high school and college. At that time, he was drinking 3 days per week, 6-8 drinks per occasion. He reported the following symptoms of dependence from this time up until about 6-7 months ago: alcohol is often taken in larger amounts or over a longer period of time than planned, a great deal of time spent drinking, drinking despite persistent the fact that it makes him feel depressed or anxious and occasionally causing blackouts, and developing tolerance (needing “a few more” drinks to get the same effect and diminished effect with same amount). He reported the following symptoms of RDC Alcoholism: 1) blackouts; 2) family objecting to his drinking (his sisters have been “displeased” on family vacations when he has gotten drunk and passed out before they were supposed to go out for dinner. The last time this happened was May 2004. He is known to get drunk at family events; “it’s expected.” 2) Others objecting to his drinking (his friends have commented when he’s “had too much”). **Alcohol Dependence, In Sustained Partial Remission is the suggested diagnosis**. **RDC Alcoholism, Past**.

Substance Use Disorders, Non-Alcohol:Subject reported the following Hallucinogen use: LSD and mescaline 2 times each in college; mushrooms once in 1993 and once in the past 2 yrs; and Extasy 1-2 times in the past yr. He denied any problems associated with this “experimental” drug use. **No diagnosis is suggested**. He reported using marijuana from his sophomore yr of high school until March 10, 2005. His use has been daily for the past 3 yrs. He stopped using marijuana on March 10, the day he called about this research study and found out that he needed to stop using in order to participate. He reported the following symptoms of dependence: substance is taken over longer period of time than planned (hrs longer); spending a great deal of time using; using the substance rather than doing important activities (such as work); continued use despite psychological problems (possibly making him feel depressed- “it sure hasn’t helped”); and developing tolerance. **Cannabis Dependence, With Physiological Dependence, in Early Full Remission is the suggested diagnosis**. He reported using cocaine during the last 2 yrs of college “a couple of weekends in a row” for a period of 6-8 weeks. He has also used cocaine in the past 2 yrs “every weekend for 4-5 weeks in a row” (multiple times using per weekend). He has used cocaine once in the past 6 months. He reported the following symptoms of dependence: substance is taken over longer period of time than planned (“before I knew it, it was 3 am”); continued use despite psychological problems (in college once his heart was racing and he was “freaked out” that something really bad might happen. Also the last time he used it in Feb, he felt “really detached”); and developing tolerance (“it’s a highly addictive substance; I’ll use whatever’s there”, using the same amount has less effect). **Cocaine Dependence, With Physiological Dependence, in Early Full Remission is the suggested diagnosis**.

Anxiety Disorders: He reported that he has felt generally anxious and uncomfortable in most social situations for as long as he can remember (definitely at least since college). He reported being afraid of “having his faults exposed and being judged unfavorably.” He reported that exposure to these situations always provokes anxiety for him; he recognizes that his fear is excessive/unreasonable (“it’s an irrational fear; I know something is wrong”). He reports avoiding social situations if he can (for example, he missed a lot of classes in college and he recently didn’t go to a work meeting). He reported that if he has to go, he can, but it is very difficult and he experiences a lot of anxiety. For example, he recently went to a co-worker’s retirement party and they were about 100 people there; he was extremely anxious. He feels that this “definitely” interferes in his life, and after the fact it bothers him a lot. **Social Phobia, Current is the suggested diagnosis**. Subject reported that during college he was afraid of going out the house alone. “Anything outside of my room was anxiety provoking and panicky; my room was my safe place.” He was afraid of having the spotlight on himself, having to prove himself. He sought out places where he could be alone: the library, a bathroom stall, wherever. He would rather be alone, because then his anxieties didn’t come up so much. This appears to be better accounted for by Social Phobia; therefore, **no diagnosis is suggested**. The subject reported “continuous” excessive worry and anxiety occurring more days than not since college. He worries about not feeling happy at work, “how I fit into things” (“the big picture”), and a lot about social situations. “I am anxious about my anxiousness.” He finds it difficult to control his worrying; “it goes haywire, the snowball effect.” He reported: restlessness and feeling keyed up or on edge, being easily fatigued, difficulty concentrating and mind going blank, irritability, muscle tension (in his shoulders), and sleep disturbance. He feels that this anxiety interferes in his life. **Generalized Anxiety Disorder, Current is the suggested diagnosis**.

Aggressive Disorders: LHA/A= 15, LHWA= 58, LHEA= 69. Subject reported that he has engaged in 40 verbal arguments and 0 temper tantrums since age 18. He reported that these began at age 12 and stopped at age 38. At their most frequent, these occurred 2 times per week for a period of 3 months at the end of his marriage. 1) The subject reported a verbal argument with his ex-wife from this period of time: he can’t remember exactly what they were disagreeing about, but he raised his voice, yelled and screamed. 2) In May of 2003, he was voicing his political views at work and raised his voice and swore. He felt shunned by his co-workers after this. 3) In October of 2004, he was “ticked off” about his work situation and raised his voice and was sarcastic to his manager. The subject reported “less than 10” incidents of minor property damage after the age of 18. He reported that this behavior started at age 13/14 and stopped at age 37. At their most frequent, these occurred at the rate of less than 2 per yr. He could only recall 2 incidents, but estimated that there might have been others. 2 yrs ago, he was frustrated about something and threw one of his plates on the ground, breaking it. He was by himself at the time. He has also done this same thing with a glass at some point. Subject reported 4-5 incidents of physical aggression, never with any injury, since age 18. These began at age 6 and stopped at age 22. He reported that growing up with his twin brother, they got in “major scuffles” about once per week. Between the ages of 18-22, these were occurring once every 3-4 months; this is the period of greatest frequency. At age 21/22, he felt that his brother was verbally accusing him of something and he reported being “on edge.” Subject started pushing and punched him in the body; his brother punched back. There were no injuries. Also at age 21/22, she wanted to borrow his sister’s TV guide, and she said something “adversarial.” He “took a swing at her” by open-fist punching her on the cheek. He reported that most of the outbursts occurred with his brother and involved shoving, pushing, or punching. While the subject’s aggression appears to cause him distress, the periods of greatest frequency occurred exclusively during periods of depression (in college at age 21/22 and at the end of his marriage). He denied ever having 2 outbursts per week for at least 1 month or 3 incidents of property damage and/or physical aggression in the same year when he was not experiencing a depressive episode. Therefore, **no diagnosis is suggested**. Suicidality/Self-Injurious Behaviors: **Suicide Attempts = 0** The subject denied any suicidal behavior, answering no when asked on the SBHF if he has ever tried to kill himself or done anything that could have killed him. **Self Injurious Behaviors = 0** The subject denied any self injurious behavior, answering no when asked on the SIBHF if he has ever tried to physically hurt himself or purposely done anything that physically harmed him.

Childhood Disorders: The subject reported that during childhood, before adolescence, others complained that he daydreamed a lot, he was easily distracted, and he tended to avoid or dislike tasks that required concentration. He reported that these problems began in 1^st^grade (age 6) and are ongoing. He reported that his teacher kept him after school for not paying attention, so it caused problems with teachers. He denied that these behaviors caused any other problems for him. **No diagnosis is suggested**. Subject reported that during childhood and adolescence he was often defiant, hostile, and negativistic towards adults. He endorsed the following symptoms during that time: often refusing to do what adults told him to do, often deliberately annoy people, often blaming others for his mistakes or misbehavior, often touchy or easily annoyed, often being angry and resentful, and often being spiteful or vindictive. These behaviors and attitudes caused problems for him at home. He reported that these behaviors occurred from around age 16 until age 18. **Oppositional Defiant Disorder, Past is the suggested diagnosis**.

**Axis II Disorders**: Subject meets General Criteria for Personality Disorder. He endorsed 38 traits as pathological with a combined score of 77.

Paranoid PD (2): *Is reluctant to confide in others because of unwarranted fear that the information will be used maliciously against him/her:* He believes there is a price to pay if he shares something personal with others, and this keeps him from confiding. He is afraid that “it might come back to haunt him” because other would use the information against him. *Reads hidden demeaning or threatening meanings into benign remarks or events:* He often reads too much into situations and takes offense at things that were not meant to be critical. His brother is often telling him that he takes things too personally. He tries to figure out what others really mean, and it sometimes turns out to be hidden threats of put downs.

Schizoid PD (1): *Lacks close friends or confidants other than first-degree relatives:* He denied having any friends that he could confide in. He wishes that he did. Schizotypal PD (3): *Ideas of reference:* He feels that though people seem to be talking in general, their comments are really meant for him. He has felt like people at work changed the rules specifically because of him several times. He sometimes feels like strangers are looking at him or talking about him. *Lacks close friends or confidants other than first-degree relatives:* He denied having any friends that he could confide in. He wishes that he did. *Suspiciousness or paranoid ideation:* See Paranoid PD section.

Borderline PD: (6): *Affective instability/marked reactivity of mood:* He reported that he has strong mood swings everyday of the week, lasting 1.5-2 hrs. He can experience the whole range of emotions. Family members and co-workers have commented that he is irritable and that his moods seem to change a great deal; he “absolutely agrees.” *Chronic feelings of emptiness:* He reported that he often feels empty; it’s always in the background. At times, this is “pretty intense.” *Transient, stress-related paranoid ideation or severe dissociative symptoms:* He reported that he has the tendency to be suspicious of people he normally trusts when under stress; “I question everything, particularly people’s motives.” This happens only when he is under stress, about once per month. *Identity disturbance: Markedly and persistently unstable self-image or sense of self:* He reported sometimes thinking about who he is and having some doubts. Although he has always considered himself heterosexual, he worries about his sexual orientation “more than I ever thought I would.” He reported that he has had 2 “past (sexual) encounters” with a male friend. This man then started calling him to presumably pursue a relationship, and the subject ignored him. *Impulsivity in at least two areas that are potentially self-damaging:* He reported the following in the past 5 yrs: been intoxicated on alcohol 30 times, been stoned or high on marijuana approximately 700 times, and driven while intoxicated 15 times. *A pattern of unstable and intense interpersonal relationships characterized by alternating between extremes of idealization and devaluation:* He reported that he doesn’t have a lot of relationships right now, but those he’s had in the past have been this way. His marriage was “very stormy”; he switched from loving someone to hating his ex wife during their marriage. **Borderline PD is the suggested diagnosis**.

Histrionic PD (2): *Self-dramatization, theatricality, and exaggerated expression of emotion:* He reports “definitely” being more expressive than most. This has caused him embarrassment and problems relating to others at work. Others have told him that he exaggerates and makes “a big deal” of things. *Considers relationships to be more intimate than they actually are:* He reported feeling a close bond with someone he has just met about once per year. He has gotten hurt in relationships because he thought it was more serious than the other person, most recently with woman from work.

Narcissistic PD (3): *Requires excessive admiration:* Other’s praise and admiration is important to him (from people in general, co-workers, friends, family). He feels hurt and empty when he doesn’t get the praise and admiration he thinks he deserves. *Believes he is special and unique and can only be understood by other special or high status people:* He has a hard time finding others like himself to share things with. He wants someone who shares the same interests, is open minded and spontaneous. They need to be special and unique also. *Sense of entitlement:* He feels “a little bit” like he has earned the right to special treatment because of past events in his life. For example, because he never really had a father, he sometimes uses that as a reason/excuse for that he should “get a break or get better treatment.”

Avoidant PD (6): 1) *Avoids occupational activities that involve significant interpersonal contact because of fears of criticism, disapproval, or rejection:* He avoids working with others because of “ a lack of comfort, confidence, and low self-esteem.” He is worried that people will criticize or reject him. 2) *Is unwilling to get involved with people unless certain of being liked:* He avoids getting to know people because he’s worried they might not like him “quite a bit of the time”; this has affected the number of friends he has. 3) *Restraint within intimate relationships owing to the fear of being shamed or ridiculed:* In close relationships, he keeps his thoughts and feelings to himself because he is afraid of being put down. “There is not questions that this kept me from sharing my true feelings a lot in my marriage.” *4) Preoccupied with being criticized or rejected in social situations:* He worries about being criticized or rejected in social situations about 45% of the time, and he can not get his mind off it (“it’s like tunnel vision”). *5) Inhibited in new interpersonal situations because of feelings of inadequacy:* He has a lot more trouble than most carrying on a conversation with someone he’s just met; he feels this is due to his social skills (or lack thereof). He is very shy and quiet because he’s afraid he might not measure up. 6*) Views self as socially inept, personally unappealing, or inferior to others:* He thinks he does not relate very well in social situations, and even when he’s at his best he feels he’s not as much fun to be around as others. He feels he is lacking in social skills. **Avoidant PD is the suggested diagnosis**.

Dependent PD (4): 1) *Has difficulty initiating projects or doing things on his own:* He usually needs help from others to get started on a project because he doesn’t feel confident in his own abilities. *2) Feels uncomfortable or helpless when alone because of exaggerated fears of being unable to care for herself:* He feel “ a lot of emptiness” when he’s alone, so much that he “gets lost in it.” He is “a little bit” afraid that he won’t be able to take care of himself emotionally. 3) *Urgently seeks another relationship as a source of care and support when a close relationship ends:* When a relationship ends, he reportedly “shuts down for awhile.” He might be “a little” desperate to get into another right away, even if it is not the best person for him. This is because he doesn’t like being alone and “it would fill the gap.” 4) *Unrealistically preoccupied with fears of being left to take care of himself:* He worries about the people he depends on leaving him, and there are times when he can’t get his mind off it. He is worried about being alone and not succeeding. He is worried that he won’t be able to take care of himself 35% of the time. Obsessive-Compulsive PD (1): *Over conscientious, scrupulous, and inflexible about matters of morality, ethics, or values (not accounted for by cultural or religious identification):* He is more strict about morals and ethics than most. He is very opposed to the war in Iraq and has “carried anger about this” and talked about it at work, which he realizes may have caused some problems for him and probably wasn’t appropriate. His co-workers have complained that he is too strict about his views and told him to “chill out.”

Self-Defeating PD (6): 1) *Chooses people and situations that lead to disappointment, failure, or mistreatment, even when better options are clearly available:* He seems to get himself in situations were he gets treated badly; he feels like this is a pattern. “Wanting my own needs fulfilled” makes it hard to avoid these situations. Most of the people he chooses to get involved with end up disappointing him or letting him down: his friends, family, romantic partners, and co-workers. 2) *Following positive personal events, responds with depression, guilt, or behavior producing pain:* When something good happens, he often finds himself felling bad dues to “a lack of full satisfaction and seeing that parts that are still missing.” He also feels like he doesn’t deserve it, and that keeps him from feeling good about. He usually expects something bad to follow something good. *3) Incites angry or rejecting responses from others and then feels hurt, defeated, or humiliated:* He often “comes off too strongly, being too much of an individual” and says things that can “come back to haunt him” by offending others. Then when other gets angry, he feels remorseful, humiliated, and hurt. *4) Rejects opportunities for pleasure, or is reluctant to acknowledge enjoying herself:* When he does something that’s fun, it’s sometimes hard to enjoy it or admit he’s had a good time, because “maybe I feel that I don’t deserve it” he turns down opportunities to have a good time “a lot.” These are things that he wants to go to, like invitations to get together after work. *5) Fails to accomplish tasks crucial to his or her personal objectives despite demonstrated ability to do so:* He “makes excuse, blames others, or is too lackadaisical” rather than doing something to actually reach his goal of moving on in his career. 6) *Is uninterested in or rejects people who consistently treat her/him well:* He consistently has trouble maintaining relationships with people who treat him better than he thinks he deserves. **Self-Defeating PD is the suggested diagnosis**.

Depressive PD (3): *Is critical, blaming, and derogatory toward self:* He has always been one to put himself down, criticize himself, and blame himself for anything that goes wrong. *Is brooding and given to worry:* He always finds something to worry about, and his brother tells him that he worries too much. He sees this as a pattern. *Is prone to feeling guilty or remorseful*: He is quick to feel guilty or responsible whenever anything goes wrong, even when others don’t blame him. He is too quick to apologize for things that aren’t his fault, and others notice this too. Negativistic PD (1): *Passively resists fulfilling routine social and occupational tasks*: he consistently does things like inventing excuses, pretending to forget, or deliberating not working very hard to get out of things he doesn’t want to do. Others are annoyed by this.

FINAL DIAGNOSES DSM-IV: Axis I: Major Depressive Episode, Recurrent Alcohol Dependence, In Sustained Partial Remission RDC Alcoholism, Past Cannabis Dependence, In Early Full Remission Cocaine Dependence, In Early Full Remission Social Phobia, Current Generalized Anxiety Disorder, Current Oppositional Defiant Disorder, Past Axis II: Avoidant Personality Disorder Borderline Personality Disorder Self-Defeating Personality Disorder GAF (average past year): 40 IED-IR: Negative MAJOR MOOD DISORDERS: Positive NIMH ALCOHOLISM: Positive DSM DRUG DEPENDENCE: Positive SUICIDAL BEHAVIOR: Negative SELF-INJURIOUS BEHAVIOR: Negative
